# Improving Freeze–Thaw Stability of High-Moisture Extruded Plant-Based Meat: A Synergistic Strategy Combining Glucose Oxidase, Phytase and Tamarind Gum

**DOI:** 10.3390/foods14244270

**Published:** 2025-12-11

**Authors:** Xuzeng Wang, Xiangquan Zeng, Jian Li

**Affiliations:** 1 National Market Supervision Administration Innovation Center (Animal Substituted Protein), Beijing Technology and Business University, Beijing 100048, China; 2 Key Laboratory of Geriatric Nutrition and Health, Ministry of Education, Beijing Technology and Business University, Beijing 100048, China; 3 Key Laboratory of Green and Low-Carbon Processing Technology for Plant-Based Food of China National Light Industry Council, School of Food and Health, Beijing Technology and Business University, Beijing 100048, China

**Keywords:** plant-based meat analogs, soy protein isolate, glucose oxidase, tamarind gum, freeze–thaw stability

## Abstract

Plant-based meat analogs, particularly those produced by high-moisture extrusion, are prone to quality deterioration during frozen storage due to poor freeze–thaw stability. This study aimed to develop a synergistic stabilization strategy for soy protein isolate (SPI)-based extrudates using glucose oxidase (GO), phytase (PA), and tamarind gum (TG). The effects of individual additives (GO, PA, TG) and their combination (GO + TG) were systematically evaluated over seven freeze–thaw cycles, with a pure soybean-protein meat analog (PSM) as a control. The results showed that repeated freeze–thaw cycles severely degraded the control groups, leading to reduced water-holding capacity (WHC), increased hardness, and color darkening. While all additives mitigated these changes, the GO + TG combination exhibited the most pronounced protective effect, maintaining the highest WHC (0.993 ± 0.000), optimal texture (hardness 66.0 ± 0.0 N, elasticity 3.7 ± 0.0 mm), and minimal color variation. Structural analyses revealed that GO + TG effectively suppressed protein oxidation, minimized sulfhydryl loss, preserved protein secondary and tertiary structures, and inhibited the conversion of immobilized water to free water. The synergistic mechanism is attributed to the formation of a dual-network structure, wherein GO enhances covalent cross-linking and TG provides steric stabilization. This study offers a practical and effective approach for enhancing the freeze–thaw stability of extruded plant-based meat products, with potential industrial applications.

## 1. Introduction

Plant-based meat analogs have garnered significant attention in recent years as sustainable alternatives to animal-derived products, driven by increasing consumer awareness of health, environmental, and ethical concerns. These products are typically formulated using plant proteins, among which soy protein isolate (SPI) is widely utilized due to its high nutritional value and functional properties, such as gelation [1] and water retention [2]. However, the structural integrity, texture, and sensory attributes of plant-based meats often fall short compared to their animal-based counterparts, particularly under processing and storage stresses [3]. To address these limitations, various enzymatic and non-enzymatic additives have been introduced to modify protein networks and enhance product quality. For instance, tamarind polysaccharide gum (TG) promotes protein cross-linking, improving sample strength and elasticity [4], while glucose oxidase (GO) enhances oxidative stability and promotes disulfide bond formation [5]. Phytase (PA) may improve protein solubility and reduce phytic acid-related negative interactions [6]. Recent studies have explored the synergistic effects of combining different additives to form multi-functional product systems with improved mechanical and stability properties [1,2,7]. Despite these advances, the behavior of such composite systems under repeated freeze–thaw conditions, a common scenario in distribution and storage, remains inadequately studied.

Freeze–thaw cycling significantly contributes to the degradation of quality in frozen foods, such as plant-based meat products. The process involves the formation and growth of ice crystals, which exert mechanical stress on the product matrix, disrupting protein networks and causing the release of immobilized water [8]. Thawing exacerbates this issue by promoting further water migration and redistribution, typically resulting in diminished water-holding capacity (WHC), increased drip loss, and textural hardening [9]. At the molecular level, freeze–thaw cycles induce changes including protein denaturation, aggregation, oxidation, and alterations in secondary and tertiary structures [10]. These changes are often evidenced by a loss of α-helix content, increased surface hydrophobicity, and the creation of carbonyl groups from oxidative side-chain reactions [11]. Furthermore, the recrystallization of ice during successive cycles amplifies these effects, causing irreversible damage to the product’s microstructure and sensory properties. Low-field nuclear magnetic resonance (LF-NMR) studies have demonstrated that transitions in the water state—specifically, the reduction in bound water (T_21_) and the rise in free water (T_23_)—are closely associated with the deterioration of product functionality [12]. Consequently, understanding the dynamics of water–protein interactions under freeze–thaw stress is essential for developing stable plant-based formulations.

Given the increasing demand for high-quality plant-based meats that withstand real-world storage conditions, there is an urgent need to develop effective strategies for improving freeze–thaw stability. While previous studies have examined the individual effects of additives such as TG or GO on protein product, few have systematically investigated their synergistic potential, particularly in combination, under multiple freeze–thaw cycles. This study aims to fill this gap by evaluating the effects of single and combined additives—GO, PA, TG, and GO + TG—on the physicochemical, structural, and stability properties of SPI-based products subjected to up to seven freeze–thaw cycles. The innovation of this work lies in its multi-scale approach, integrating macro-scale texture and color measurements with micro- and molecular-level analyses, including particle size, zeta potential, FTIR, fluorescence spectroscopy, LF-NMR, surface hydrophobicity, and protein oxidation markers. Furthermore, molecular dynamics simulations are employed to elucidate the stabilizing mechanisms of tamarind gum–protein interactions at the atomic level, providing theoretical insights into the structural stabilization observed experimentally. By demonstrating the synergistic efficacy of GO and TG in preserving sample integrity, water distribution, and protein conformation, this research offers a scientifically grounded strategy for enhancing the freeze–thaw stability of plant-based meat products, thereby contributing to the development of more resilient and consumer-acceptable analog foods.

## 2. Materials and Methods

### 2.1. Materials

**Figure 1:**
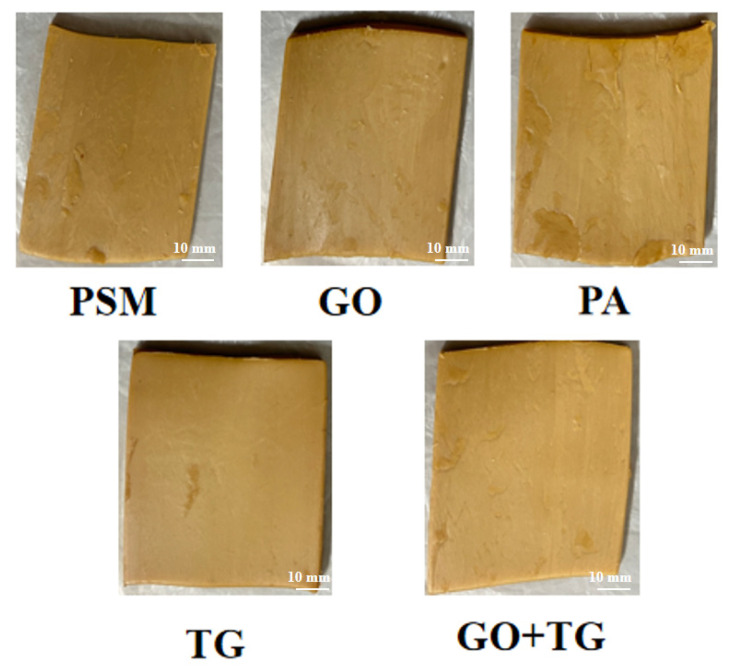
Images of extruded products of plant-based meat PSM, GO, PA, TG, and GO + TG. Note: PSM, GO, PA, and TG, respectively, represent pure soybean meat, glucose oxidase, phytase, and tamarind gum. The scale bar on the photo is 10 mm.

The plant-based meat analogs (Figure 1) were prepared using soy protein isolate (SPI) as the primary ingredient, sourced from Shanghai Yuanye Bio-Technology Co., Ltd. (Shanghai, China). Glucose oxidase (GO, 250 U/mg) and tamarind gum (TG) were procured from Sigma-Aldrich (St. Louis, MO, USA). Phytase (PA, 200 U/g) was obtained from Sinopharm Chemical Reagent Co., Ltd. (Shanghai, China). All other reagents utilized in this study were purchased from Guojun Group Chemical Reagents Co., Ltd. (Dezhou, China). All chemical reagents employed were of analytical grade.

### 2.2. Preparation of Plant-Based Meat with Different Additives

The basic plant-based meat mixture (PSM) was prepared by mixing soy protein isolate (SPI, 98% *w*/*w*) with deionized water (100%, *v*/*v*) at a ratio of 1:7 (weight/volume). The treatment groups were prepared [13] by incorporating 2‰ (*w*/*w*, based on total mixture) of glucose oxidase (GO, E.C. 1.1.3.4), 2‰ (*w*/*w*, total mixture) of phytase (PA, E.C. 3.1.3.2), 3.5% (*w*/*w*, total mixture) of tamarind gum (TG), or a combination of 2‰ GO and 3.5% TG into the PSM mixture. The mixtures were homogenized at 10,000 rpm for 5 min, then placed in deionized water (60 °C), stirred at 120 rpm for 60 min, and texturized using a twin-screw high-moisture extruder (SPX-50, Shandong Yuwang Ecological Food Industry Co., Ltd., Dezhou, China). The extrusion process parameters were set as follows: the screw speed was 250 rpm, the temperatures of the five barrel zones from the feeding end to the die end were controlled at 60 °C, 90 °C, 120 °C, 150 °C, and 180 °C, respectively, and the cooling die temperature was maintained at 70 °C. Each kilogram of soy protein can produce approximately 7 kg of plant-based meat. The products were then cooled to room temperature; then, the plant-based meat was cut into pieces measuring 3 cm × 3 cm × 5 mm and stored in sealed plastic containers at 4 °C for further analysis. Samples stored at 4 °C can be kept for a maximum of 24 h.

### 2.3. Freeze–Thaw Treatment

The samples (3 cm × 3 cm × 5 mm) were individually sealed in plastic containers to prevent moisture loss and surface dehydration. The samples were subjected to freeze–thaw cycles as follows: freezing at −20 °C for 20 h in a conventional freezer (451L, Haier, Qingdao, China), followed by thawing at 4 °C for 4 h in a temperature-controlled incubator (XM-18, Feinade, Kaifeng, China). The thawing process was considered complete when the center temperature of the sample reached 4 °C, monitored using a needle-type thermocouple (PT100E, Wanping, Yangzhou, China). Samples were subjected to 1, 2, 3, 4, 5, 6, or 7 complete freeze–thaw cycles. The entire procedure was performed in triplicate for each treatment group using independently prepared batches. Samples collected prior to any freeze–thaw treatment served as the untreated control. After each specified number of cycles, samples were collected for subsequent analysis. Some of the samples were dried using a freeze dryer (ZL-20D, Zhongeling Technology, Guangzhou, China) and then stored in the form of freeze-dried powder. This cycle was selected to simulate common freeze–thaw conditions encountered during the distribution and storage of frozen plant-based products, as referenced in previous studies [8,9,12].

### 2.4. Carbonyl Content Analysis

Carbonyl content was detected using the freeze-dried powder, as described in Section 2.3. Carbonyl content [14] was assessed using the 2,4-dinitrophenylhydrazine (DNPH) method. Protein samples were subjected to a reaction with DNPH, and the absorbance was measured at 370 nm. The carbonyl content was then calculated utilizing a molar extinction coefficient of 22,000 M^−1^·cm^−1^. Each group of experiments was repeated three times.

### 2.5. Free and Total Sulfhydral Group Analysis

A certain mass of the plant meat sample was weighed and added to the pre-cooled Tris-Glycine buffer solution (0.086 M Tris, 0.09 M Glycine, 4 mM EDTA, pH 8.0) at a 1:10 (*w*/*v*) ratio. Under ice-water bath conditions, a high-speed homogenizer was used to thoroughly homogenize the mixture to ensure the effective extraction of proteins while preventing overheating. Subsequently, the homogenate was centrifuged at 4 °C and 10,000× *g* for 20 min. After centrifugation, the supernatant was carefully collected, serving as the protein extraction solution. Free and total sulfhydral groups [14] were quantified using Ellman’s reagent (DTNB). To measure free sulfhydral groups, samples were combined with Tris-Gly-EDTA buffer and DTNB, and the absorbance was measured at 412 nm. Total sulfhydral groups were determined following incubation with urea and SDS. The results are presented as mg/g protein. Each group of experiments was repeated three times.

### 2.6. Surface Hydrophobicity Analysis

The plant-based meat samples were weighed, and pre-cooled phosphate buffer (0.01 M, pH 7.0) was added at a ratio of 1:15 (*w*/*v*). High-speed homogenization was carried out under ice bath conditions to obtain a protein suspension. Subsequently, the suspension was centrifuged at 10,000× *g* and 4 °C for 15 min, and the supernatant was collected as the protein extraction stock. The original solution was diluted step by step using the same phosphate buffer to prepare a series of protein solutions of different concentrations. All diluted samples must be immediately used for ANS binding experiments and fluorescence intensity determination after preparation to accurately reflect the surface properties of proteins in their natural state. The surface hydrophobicity [15] was determined using the fluorescent probe 1-anilinonaphthalene-8-sulfonate (ANS). The samples were diluted to a protein concentration of 1 mg/mL, then mixed with the ANS solution and incubated for 10 min. The fluorescence intensity was measured at an excitation wavelength of 390 nm and an emission wavelength of 470 nm. Each group of experiments was repeated three times.

### 2.7. Tertiary Structure Analysis

The phosphate-buffered solution was prepared using the method described in Section 2.6. The protein extract was diluted appropriately with the same buffer to ensure that its absorbance value at a wavelength of 280 nm was less than 0.1, thereby effectively avoiding any spectral distortion that may be caused by the fluorescence internal filtration effect. If the diluted solution was slightly turbid, it had to be centrifuged again at 10,000× *g* for 10 min at 4 °C to obtain a clear and transparent supernatant for spectral scanning. The final sample was collected for fluorescence emission spectra at an excitation wavelength of 280 nm. The tertiary structure of proteins was assessed using intrinsic fluorescence spectroscopy (F-7000, Hitachi, Tokyo, Japan). The excitation wavelength was set to 295 nm [16], and the emission spectrum was recorded between 300 and 400 nm. The maximum fluorescence intensity and emission wavelength were documented. Each group of experiments was repeated three times.

### 2.8. Secondary Structure Analysis

The secondary structure analysis was conducted using the freeze-dried powder as described in Section 2.3. Fourier transform infrared (FTIR) [16] spectroscopy (specifically the Nicolet iS50 model from Thermo Fisher Scientific, Waltham, MA, USA) was utilized to analyze the secondary structure of proteins. FTIR spectra were collected over a range of 4000–400 cm^−1^ with 32 scans per spectrum at a resolution of 4 cm^−1^. The amide I band, ranging from 1600 to 1700 cm^−1^, was deconvoluted and fitted to quantify the percentages of α-helix, β-sheet, β-turn, and random coil components. Each group of experiments was repeated three times.

The spectral analysis procedure was performed as follows: a linear baseline was applied between 1700 and 1600 cm^−1^, and the amide I band was deconvoluted using a Gaussian function with a half-bandwidth of 25 cm^−1^ and an enhancement factor of 2.5 to resolve the underlying component bands. Then, second-derivative spectroscopy was applied to identify the number and approximate positions of these component bands. Finally, a curve-fitting procedure was performed on the original (non-deconvoluted) amide I band using a combination of Gaussian and Lorentzian functions (70:30) to quantitatively estimate the relative percentages of different secondary structures. The areas of the fitted bands were assigned to specific structural elements as follows: 1650–1660 cm^−1^ for α-helix, 1610–1640 cm^−1^ for β-sheet, 1660–1680 cm^−1^ for β-turn, and 1640–1650 cm^−1^ for random coil. The relative content of each secondary structure was calculated as the percentage ratio of the area of its corresponding band to the total area of the amide I band. The curve-fitting process was iterated until a convergence criterion of R^2^ > 0.99 was achieved.

### 2.9. Low-Field Nuclear Magnetic Resonance (LF-NMR) Analysis

LF-NMR testing was performed using the freeze-dried powder as described in Section 2.3. LF-NMR measurements [12] were conducted using a NMI20 analyzer from Niumag Corporation, Suzhou, China. The transverse relaxation time (T_2_) was determined using the Carr–Purcell–Meiboom–Gill (CPMG) sequence. The relative proportions of bound water (T_21_), immobilized water (T_22_), and free water (T_23_) were then calculated.

### 2.10. Water-Holding Capacity (WHC) Analysis

After each freeze–thaw cycle, samples were immediately taken for water-holding capacity (WHC), texture profile analysis (TPA), and colorimetric tests. Water-holding capacity (WHC, %) was determined [17] by centrifuging 5 g of sample at 10,000× *g* for 15 min at 4 °C. The WHC was calculated as the ratio of the sample’s weight post-centrifugation to its initial weight. Each group of experiments was repeated three times.

### 2.11. Texture Profile Analysis (TPA)

We used a texture analyzer (TA.XT Plus, Stable Micro Systems, London, UK) equipped with a P/50 probe to measure the hardness (N), elasticity (mm), and chewability (mJ) of the sample. The test conditions were as follows: pre-test speed of 2.0 mm/s, test speed of 1.0 mm/s, post-test speed of 2.0 mm/s, compression strain of 50%, and a trigger force of 5 g. Each sample was measured nine times.

### 2.12. Colorimetric Analysis

Color parameters (L*, a*, b*) were measured using a chromameter (CR-400, Konica Minolta, Tokyo, Japan). Prior to use, the instrument was calibrated with a white standard plate. Measurements were taken at five random locations on each sample’s surface, with 3 samples measured for each group of experiments.

### 2.13. Molecular Dynamics Simulation

The stability and kinetic properties of the simulated protein–ligand complex in an aqueous environment were examined using the Gromacs 2022 dynamics simulation software and the Amber99sb-ildn force field for protein–small molecule interactions. A 13 × 13 × 3 nm^3^ ice surface was constructed using Genice. The system was hydrated with the TIP4P water model, and a water box measuring 13 × 13 × 10 nm^3^ was created (ensuring that the edges of the water box were at least 1.2 nm from the protein’s edges). An automatically balancing system was implemented.

Protein model selection and preparation [18]: the representative protein model selected for the simulation was the Glycinin (11S) A3B4 subunit homohexamer (PDB ID: 1OD5). Glycinin is the most abundant storage protein in soy protein isolate (SPI), and the A3B4 subunit is a predominant form. Its well-characterized three-dimensional structure makes it a suitable and widely adopted model for simulating SPI behavior in molecular studies. The structure was retrieved from the Protein Data Bank, and any missing residues or atoms were reconstructed and protonated according to physiological pH (pH 7.0) using UCSF Chimera (v 1.17.3).

Ligand (TG) model selection and preparation [19]: the ligand in this simulation was a representative oligosaccharide unit of tamarind gum (TG). TG is a high-molecular-weight polysaccharide composed of a cellulose-like backbone (β-1,4-glucose) with xylose and galactoxylose side chains. A characteristic repeating unit with the sequence GXXLGXXXGG was modeled, where G represents a glucose unit in the backbone, X represents a glucose unit substituted with an α-(1,6)-linked xylose, and L represents an X unit further substituted with a β-(1,2)-linked galactose on the xylose.

### 2.14. Statistical Analysis

The results were presented as the mean ± standard deviation (SD). Statistical data analysis and graphing were performed using Origin software (version 2022, OriginLab, Northampton, MA, USA). A one-way analysis of variance (*p* < 0.05) was employed to identify significant differences among the samples, and the Tukey test was used to distinguish the mean values of the datasets.

## 3. Results and Discussion

### 3.1. Analysis of Carbonyl Content

**Figure 2:**
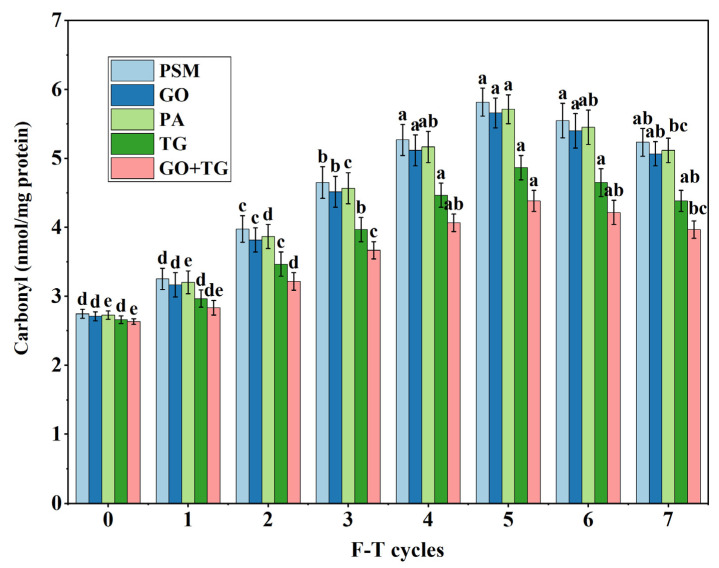
The effect of 7 freeze–thaw cycles on the carbonyl content in plant-based meat. Note: PSM, GO, PA, and TG, respectively, represent pure soybean meat, glucose oxidase, phytase and tamarind gum. Values are mean ± SD; different superscript letters within a column indicate significant differences (*p* < 0.05).

The content of carbonyl groups serves as a direct indicator of protein oxidative damage. An increase in this content suggests that amino acid side chains (such as those of lysine and arginine) are being attacked by reactive oxygen species to form carbonyl derivatives [20]. The higher the value in units of nmol/mg protein, the more severe the oxidation. The carbonyl content in all groups significantly increases (Figure 2). In the absence of freeze–thaw cycles, the PSM group exhibits a carbonyl content ranging from 2.68 to 2.81 nmol/mg. Following seven freeze–thaw cycles, this value rises to between 5.05 and 5.45 nmol/mg. However, after the same number of freeze–thaw cycles, the GO + TG group’s carbonyl content reaches only 3.98 ± 0.06 nmol/mg, indicating the smallest increase. Based on the order of groups (GO + TG < TG < GO < PA < PSM), it is evident that GO + TG can effectively inhibit protein oxidation. Repeated phase changes and ice crystal formation facilitate the release of metal ions and lipid peroxidation, generating free radicals that target proteins [21]. GO catalyzes the oxidation of glucose to consume oxygen and reduce oxidized substrates [22]; TG hinders the propagation of free radicals by chelating metal ions and providing a physical barrier effect [23]. The combination of these two substances creates a multi-faceted protective mechanism, markedly decreasing the rate of carbonyl formation. These findings support the mechanism of freeze–thaw-induced protein oxidation [24] and demonstrate the efficacy of a combined antioxidant strategy for application in plant-based meat products.

### 3.2. Analysis of Free Sulfhydral Groups and Total Sulfhydral Groups

**Figure 3:**
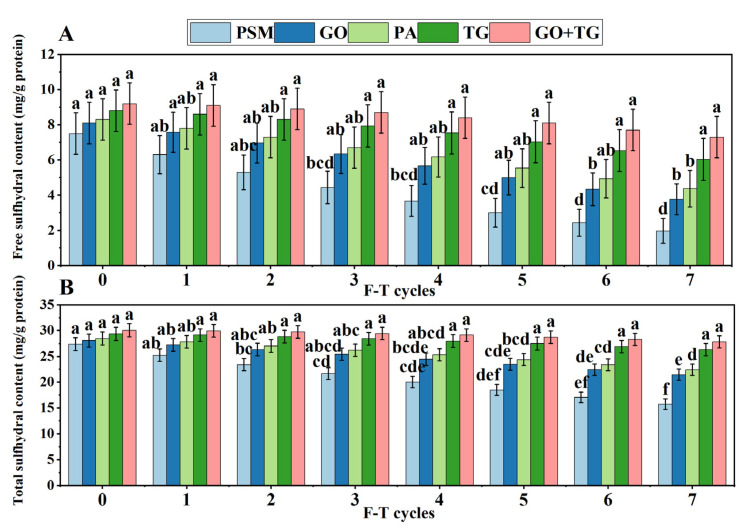
The effect of 7 freeze–thaw cycles on the free and total sulfhydral content in plant-based meat. (**A**) Free sulfhydral contents; (**B**) Total sulfhydral contents. Note: PSM, GO, PA, and TG, respectively, represent pure soybean meat, glucose oxidase, phytase, and tamarind gum. Values are mean ± SD; different superscript letters within a column indicate significant differences (*p* < 0.05).

Free sulfhydral groups reflect the number of active sulfhydral groups in the protein molecule, and their decrease often indicates oxidation or the formation of disulfide bonds. Total sulfhydral groups include both free and bound forms, and their reduction signifies an increase in the overall oxidation level of the protein [25]. The levels of free sulfhydral groups and total sulfhydral groups in all groups continue to diminish (Figure 3A,B) with an increase in freeze–thaw cycles. After seven freeze–thaw cycles, the free sulfhydral groups in the PSM group decreased to 1.97 ± 0.02 mg/g and the total thiol groups to 15.73 ± 0.05 mg/g, whereas in the GO + TG group, the free sulfhydral groups were 7.3 ± 0.03 mg/g and the total sulfhydral groups were 27.8 ± 0.07 mg/g, with the least reduction (*p* < 0.05). This indicates that the additives, particularly GO + TG, can effectively protect sulfhydral groups from oxidation.

During the freeze–thaw process, the formation and recrystallization of ice crystals cause mechanical stress, which promotes the unfolding of proteins and the exposure of sulfhydral groups, leading to their oxidation into disulfide bonds or other derivatives [26]. GO reduces the oxidative environment by consuming oxygen, while TG inhibits protein aggregation through spatial steric hindrance. Together, they synergistically delay the loss of sulfhydral groups. The decrease in total sulfhydral groups further confirms the oxidation of the protein main chain and the formation of carbonyl compounds (as shown in Section 3.9). Our results corroborate the mechanism of freeze–thaw-induced protein oxidation [27] and underscore the advantage of using composite additives to maintain protein structural integrity.

### 3.3. Surface Hydrophobicity Results

Surface hydrophobicity is a critical parameter for assessing the extent of exposure of hydrophobic regions within protein molecules. An elevation in this value generally signifies protein denaturation, aggregation, or unfolding [28]. In this study, hydrophobicity was measured using a fluorescence probe technique. The greater the value, the more severe the structural damage to the protein [29].

**Figure 4:**
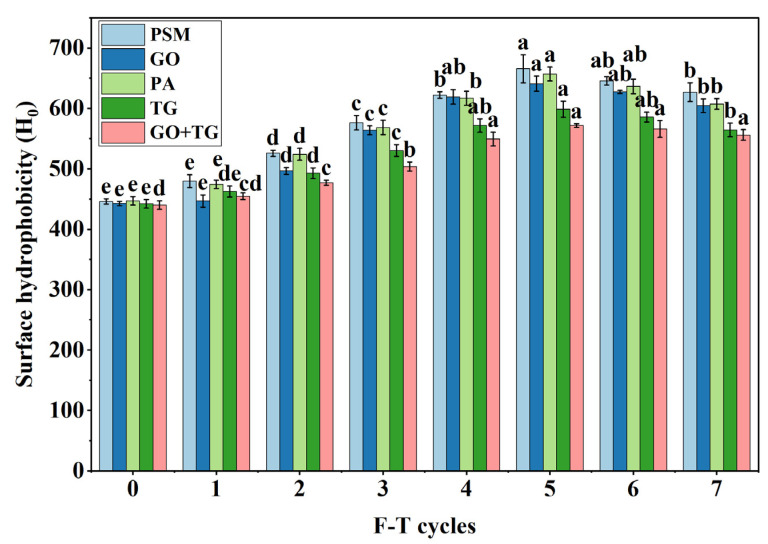
The effect of 7 freeze–thaw cycles on the surface hydrophobicity in plant-based meat. Note: PSM, GO, PA, and TG, respectively, represent pure soybean meat, glucose oxidase, phytase, and tamarind gum. Values are mean ± SD; different superscript letters within a column indicate significant differences (*p* < 0.05).

The surface hydrophobicity of all groups significantly increased (Figure 4) as the number of freeze–thaw cycles rose. Before freeze–thaw cycles, the PSM group’s value ranged from 448 to 441, but after seven freeze–thaw cycles, it increased to between 611 and 642. In contrast, the GO + TG group’s value only rose to between 550 and 566 after seven freeze–thaw cycles, showing the smallest increase. There were significant differences among the groups: GO + TG < TG < GO < PA < PSM. For instance, the TG group’s value was between 552 and 574, the GO group’s value was between 595 and 617, and the PA group’s value was between 598 and 616. This indicates that GO + TG can effectively inhibit the exposure of protein hydrophobic groups. The growth of ice crystals and phase transitions cause the disruption of intermolecular forces between protein molecules, exposing hydrophobic regions, which subsequently leads to aggregation [30]. GO forms disulfide bonds through enzymatic reactions, strengthening the protein network [31]; TG covers the exposed sites through hydrogen bonds and hydrophobic interactions, reducing aggregation [32]. When GO and TG are used in combination, their synergistic effect further stabilizes the protein conformation and delays the increase in hydrophobicity. This observation is in agreement with the findings [33] reported that polysaccharide–enzyme complexes can inhibit freeze–thaw denaturation, and it further confirms the established negative correlation between surface hydrophobicity and protein functional properties.

### 3.4. Analysis of Tertiary Structure

The endogenous fluorescence spectrum, particularly that of tryptophan residues, serves as a sensitive indicator for detecting alterations in the tertiary structure of proteins. Upon denaturation of proteins, tryptophan residues, which are typically embedded within the molecule’s hydrophobic core, become exposed to a hydrophilic environment. This exposure generally leads to an increase in their maximum fluorescence intensity and/or a shift in the emission wavelength, which may be a redshift or a blueshift [34].

**Figure 5:**
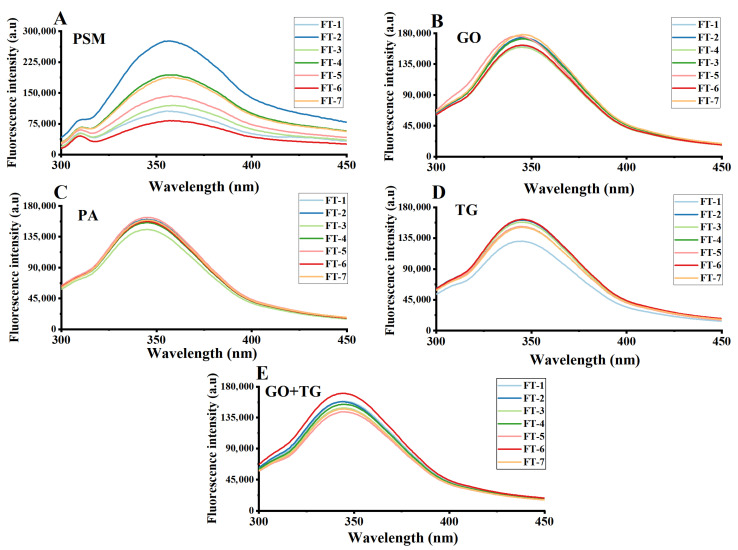
The effect of 7 freeze–thaw cycles on the endogenous fluorescence spectra on plant-based meat. (**A**) PSM group; (**B**) GO group; (**C**) PA group; (**D**) TG group; (**E**) GO + TG group. Note: PSM, GO, PA, and TG, respectively, represent pure soybean meat, glucose oxidase, phytase, and tamarind gum.

As the number of freeze–thaw cycles increases, the fluorescence intensity of all samples (Figure 5) within the 330–350 nm characteristic wavelength range exhibits an upward trend. The fluorescence intensity of the PSM group (Figure 5A) increased the most, which strongly indicates that the protein structure of this group underwent the most extensive unfolding. A large number of originally encapsulated tryptophan residues were structurally disrupted and exposed in the aqueous phase [35]. This unfolding of the structure is one of the fundamental reasons for the deterioration of the functional properties of proteins (such as water-holding capacity).

The fluorescence intensity increase in the treatment group (Figure 5B–E) was relatively gentle, suggesting that it has a certain protective effect on the tertiary structure of proteins [35]. Among them, the GO + TG compound group (Figure 5E) exhibited the most stable fluorescence signal, with minimal variation. This reveals its outstanding protective mechanism: the TG forms a physical barrier around the protein molecule through its high molecular chain wrapping, directly reducing the contact between the tryptophan residues and the external hydrophilic environment [36]. More importantly, the protein cross-linking reaction catalyzed by GO tightens the three-dimensional structure of the protein from the inside, making it more difficult for the protein sphere to expand under freezing and thawing stress and effectively locking the tryptophan residues in the hydrophobic core [37]. This external wrapping and internal tightening collaborative strategy enables the GO + TG group to most effectively maintain the natural tertiary structure of proteins and inhibit the fluorescence enhancement effect caused by freezing and thawing.

### 3.5. Secondary Structure Analysis Results

Infrared spectroscopy is a powerful tool for analyzing changes in the secondary structure of proteins. By performing deconvolution fitting on the amide I band (ranging from 1600 to 1700 cm^−1^), the relative contents of the α-helix, β-sheet, β-turn, and random coil can be quantified. Protein denaturation is typically characterized by the transformation from ordered structures (such as the α-helix) to disordered structures (like the β-turn and random coil) [38].

**Figure 6:**
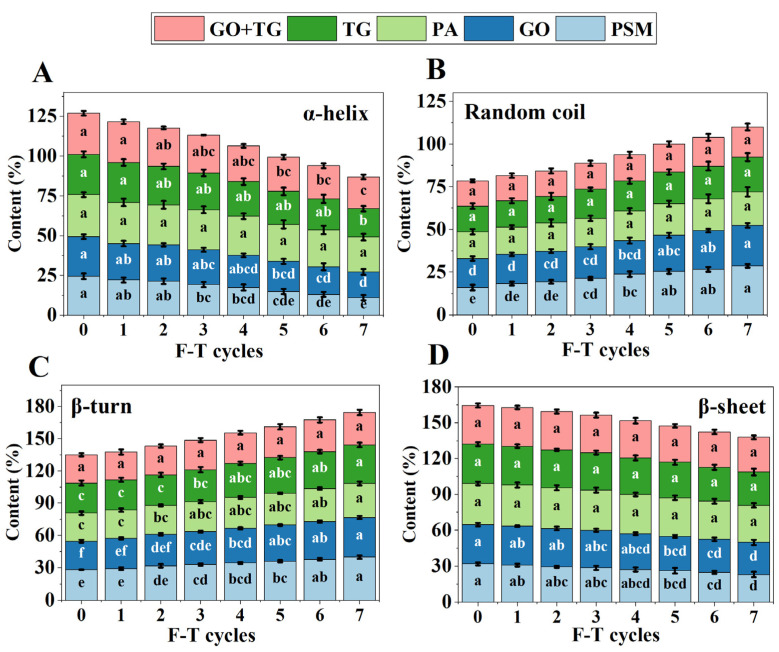
The effect of 7 freeze–thaw cycles on the Fourier transform infrared (FTIR) spectroscopy of plant-based meat. (**A**) α-helix contents; (**B**) Random coil contents; (**C**) β-turn contents; (**D**) β-sheet contents. Note: PSM, GO, PA, and TG, respectively, represent pure soybean meat, glucose oxidase, phytase, and tamarind gum. Values are mean ± SD; different superscript letters within a column indicate significant differences (*p* < 0.05).

The α-helix content (Figure 6A) in the PSM group decreased sharply from 24.2 ± 0.8% for no freeze–thaw cycles to 11.4 ± 0.8% for seven freeze–thaw cycles. Simultaneously, the random coil content (Figure 6B) increased from 15.7 ± 0.8% to 28.5 ± 0.5%. This dramatic structural change clearly indicates that freeze–thaw stress causes the highly ordered helical structure of soy protein to unravel, with the molecular chains becoming loose and disordered, which is direct evidence of severe protein denaturation [39].

The presence of additives significantly slowed down the denaturation process to varying degrees. The TG group exhibited a certain protective effect, as its colloid network could reduce local stress on the protein structure by restricting the migration of water molecules and the macroscopic growth of ice crystals [4]. However, the most effective protection was provided by the GO + TG combination group. The α-helix content of this group decreased the least, from 26.2 ± 0.7% to 19.7 ± 0.7%. Its protective mechanism was synergistic: the active intermediates generated by GO catalysis could induce the formation of additional disulfide bonds between protein molecules [40]. This covalent cross-linking stabilized and fixed the natural conformation of the protein, particularly the α-helix structure, making it less susceptible to unwinding under freezing and thawing stress [41]. TG provided a stable macroscopic microenvironment, while GO reinforced the protein’s internal structure. Thus, their combination most effectively maintained the integrity of the protein’s high-level structure.

### 3.6. Low-Field Nuclear Magnetic Analysis

Low-field nuclear magnetic resonance (LF-NMR) measures the relaxation time (T_2_) of water molecules and their relative distribution (P_2_) to reflect the state and fluidity of water in food systems. Bound water (T_21_) is closely associated with proteins and is not easily mobile; water existing in the product network is less mobile (T_22_), while free water (T_23_) is easily lost [42]. The relative area changes of these water types can directly indicate the water-holding capacity and stability of the product.

As the number of freeze–thaw cycles increased, the relative area of bound water (P_21_) in all samples exhibited a downward trend, while the relative area of free water (P_23_) significantly increased. This indicates that the freeze–thaw process disrupted the protein–water interaction, resulting in the redistribution of water [43]. Taking seven freeze–thaw cycles as an example (Table 1), the P_21_ of the PSM group was only 496.06, and the P_23_ was as high as 227.02. In contrast, the P_21_ of the GO + TG group was 976.96, and the P_23_ was 101.23. This demonstrates that GO + TG can effectively inhibit water migration. A comparison between groups revealed that the GO + TG group maintained the highest P_21_ and the lowest P_23_ at all freeze–thaw stages, followed by the TG group (P_21_: 905.67, P_23_: 127.75), the GO group (P_21_: 850.31, P_23_: 182.14), the PA group (P_21_: 880.19, P_23_: 201.91), and the PSM group, which performed the worst. This suggests that the synergistic effect of GO and TG can enhance the protein network structure, reduce the compression of ice crystals on water, and thereby improve water retention. This phenomenon can be attributed to the hydrogen peroxide produced by GO [22], which promotes protein cross-linking, and TG, as a hydrophilic colloid, enhances water interaction, jointly stabilizing the water phase state [44]. Furthermore, the LF-NMR parameters are closely related to the texture characteristics, and a high P_21_ value typically corresponds to better product texture [45]. The results of this study are consistent with the aforementioned literature, confirming that the GO + TG composite additive has significant advantages in the freeze–thaw stability of plant-based meat.

### 3.7. Water-Holding Capacity

The water-holding capacity (WHC) directly reflects the integrity and stability of the product network structure [46]. A decrease in the WHC value typically indicates that the network structure has been compromised during freeze–thaw processes, leading to water loss and a deterioration in texture.

**Figure 7:**
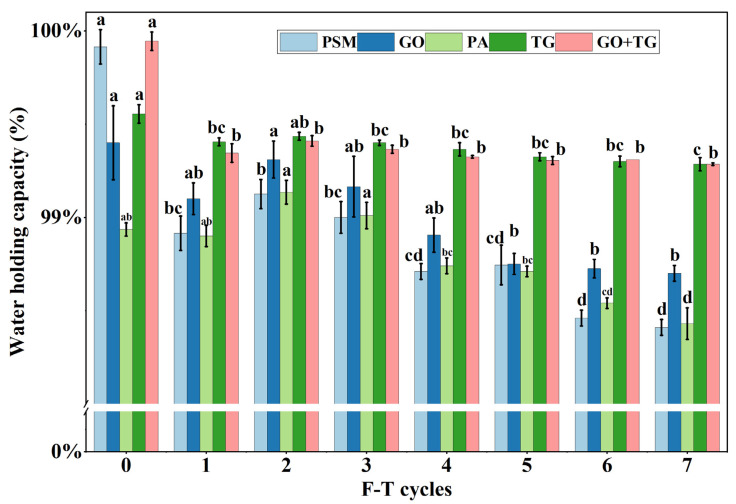
The effect of 7 freeze–thaw cycles on the water-holding capacity (WHC) on plant-based meat. Note: PSM, GO, PA, and TG, respectively, represent pure soybean meat, glucose oxidase, phytase, and tamarind gum. Values are mean ± SD; different superscript letters within a column indicate significant differences (*p* < 0.05).

As the number of freeze–thaw (F-T) cycles increases (from the control group to seven times), the water-holding capacity (WHC) value of all experimental groups exhibits a systematic decline (Figure 7). This is primarily due to the formation, growth, and recrystallization of ice crystals during repeated freeze–thaw processes. The ice crystals continuously exert mechanical puncturing and squeezing on the protein network, causing the network structure to become loose and the water retention cavities to collapse, thereby forcing the bound water and some free water to be released [47]. The PSM group (Figure 7) decreased significantly from 0.9992 ± 0.0003 (0 cycles) to 0.9841 ± 0.0003 (7 cycles), with the most drastic decline, indicating that its network is the most sensitive to freeze–thaw stress. The WHC values in the PSM and PA groups slightly increased after the second freeze–thaw cycle. This might be due to the temporary reorganization of the structure network. During the early freeze–thaw cycles, the formation of ice crystals could have caused the network to temporarily contract, resulting in a slight increase in WHC. However, as the cycles continued, structural damage dominated, causing WHC to decrease.

In contrast, all the treatment groups demonstrated varying degrees of protective effects. Among these, the group that combined TG with GO exhibited the most outstanding performance. The WHC value decreased only slightly, from 0.999 ± 0.0002 to 0.9928 ± 0.0001. This phenomenon can be attributed to the synergistic enhancing effect between TG and GO. As a hydrophilic colloid, TG can construct a hydrophilic network throughout the system via hydrogen bonds and molecular entanglements. This network not only holds a large amount of water itself but also acts as a framework to enhance the rigidity of the protein network, effectively buffering the damage caused by ice crystals [48]. GO may moderately reduce the local pH of the system by catalyzing glucose to form gluconic acid and hydrogen peroxide or promote the formation of disulfide bonds between protein molecules through oxidation, thereby strengthening the covalent cross-linking network of the protein [5]. The synergistic effect of the two constructs forms a more compact and stable composite system, significantly delaying the loss of water retention.

### 3.8. Texture Characteristic Analysis

Texture is a key sensory attribute for evaluating the eating quality of plant-based meat. Among these, hardness, elasticity, and chewiness are the core parameters. Freezing and thawing cycles often lead to deterioration of the product’s texture, such as increased hardness and loss of elasticity [49], which is closely related to the physical damage of muscle fiber protein structure by ice crystals and the aggregation of protein denaturation.

**Figure 8:**
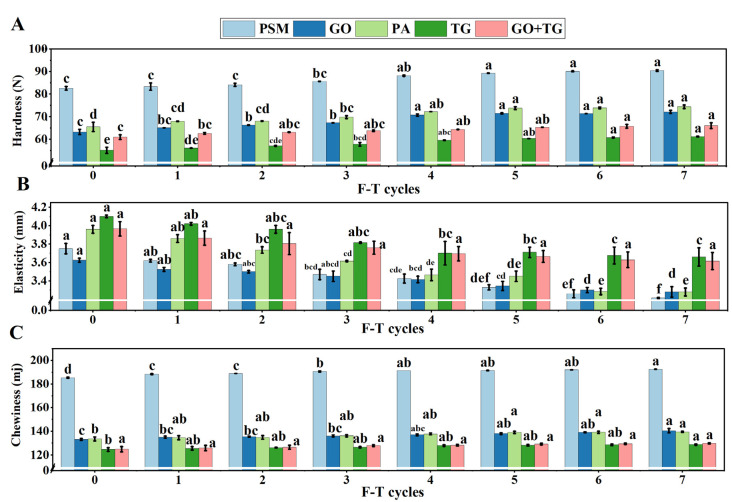
The effect of 7 freeze–thaw cycles on the texture profile analysis (TPA) of plant-based meat. (**A**) Hardness value; (**B**) Elasticity value; (**C**) Chewiness value. Note: PSM, GO, PA, and TG, respectively, represent pure soybean meat, glucose oxidase, phytase, and tamarind gum. Values are mean ± SD; different superscript letters within a column indicate significant differences (*p* < 0.05).

The data from this study clearly illustrate the deterioration process. The hardness (Figure 8A) of the PSM group increased significantly from 82.60 ± 0.29 N in the control group to 90.40 ± 0.14 N after seven cycles, while the elasticity (Figure 8B) decreased from 3.75 ± 0.02 mm to 3.22 ± 0.00 mm. This indicates that its internal structure became more compact and rigid under freeze–thaw stress, but the recovery ability weakened. The hardness increase of the GO group and the PA group was smaller than that of the PSM group, suggesting that enzyme treatment may maintain structural flexibility to some extent by modifying protein molecules [50] (such as the oxidation cross-linking of GO or the hydrolysis of the phosphate groups of PA). The chewing changes (Figure 8C) were consistent with the trends of hardness and elasticity, further confirming the negative impact of freeze–thaw cycles on the edible quality of the product. The synergistic effect of GO and TG not only optimizes the hardness and elasticity but also jointly endows the product with a more pleasant chewing characteristic, avoiding the texture hardening caused by freeze–thaw cycles.

However, the group containing TG performed the best. Throughout the entire freeze–thaw process, the TG group maintained the lowest hardness (61.19 ± 0.06 N after seven freeze–thaw cycles) and the highest elasticity (3.66 ± 0.04 mm after seven freeze–thaw cycles). This is due to the viscoelastic network formed by the long-chain molecules of TG, which can effectively absorb and disperse the stress generated during freeze–thaw cycles, acting like a molecular spring, thereby protecting the protein structure from irreversible damage and maintaining the soft and juicy texture of the product [51]. Particularly important is the combination group of TG and GO, which achieved the best balance between hardness and elasticity. Its hardness (66.04 ± 0.46 N after seven cycles) was slightly higher than that of the only-TG group, but the elasticity remained excellent. This may be due to the mutual penetration of the covalent protein network formed by GO promotion and the physical entanglement network of TG [52], forming a more stable and resilient dual-network structure [53]. This structure can resist deformation and recover quickly after stress removal.

### 3.9. Color Parameters

Color plays a crucial role in shaping consumers’ initial perception of plant-based meat products. The reduction in L* value (indicating brightness) and the rise in a* value (indicating redness) and b* value (indicating yellowness) are typical signs of color degradation during freezing and thawing processes [54]. These changes are primarily associated with alterations in light scattering due to water migration, fat oxidation, and the buildup of Maillard reaction products [55].

**Figure 9:**
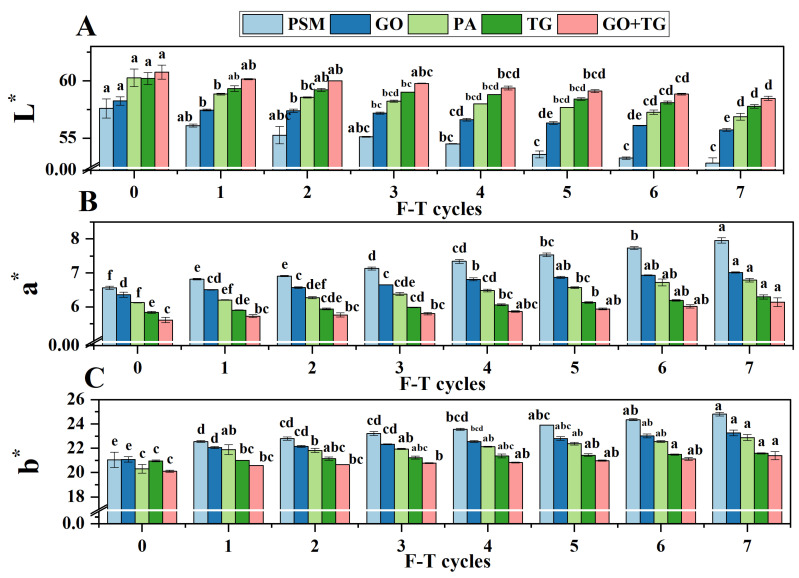
The effect of 7 freeze–thaw cycles color changes on plant-based meat. (**A**) L* value; (**B**) a* value; (**C**) b* value. Note: PSM, GO, PA, and TG, respectively, represent pure soybean meat, glucose oxidase, phytase and tamarind gum. Values are mean ± SD; different superscript letters within a column indicate significant differences (*p* < 0.05).

After seven freeze–thaw cycles, all groups exhibited a pattern of diminished L* values (Figure 9A) and elevated a* (Figure 9B) and b* (Figure 9C) values. The PSM group experienced the most pronounced shifts: the L* value dropped from a range of 57.60 ± 0.30 to 52.83 ± 0.17; the a* value rose from 6.57 ± 0.02 to 7.96 ± 0.03; and the b* value increased from 21.05 ± 0.23 to 24.81 ± 0.05. This suggests that the surface color became notably darker, with intensified red and yellow hues, leading to a decline in sensory quality. The application of additives, to varying degrees, delayed these color changes.

Among them, the color protection effect of the group combining TG with GO was the most significant. The L* value remained at a high level throughout (it was still 58.47 ± 0.07 after seven cycles), and the growth rates of a* and b* values were the smallest. This outstanding color protection performance can be explained from two perspectives: Firstly, the dense network formed by TG can effectively lock in moisture, reducing the light scattering changes caused by water evaporation or surface drying, thereby maintaining the brightness of the product surface (L* value) [56]. Secondly, GO consumes oxygen in the system and may change the reactivity of reducing sugars and amino acids through oxidation, effectively inhibiting the processes of lipid oxidation and the Maillard reaction, which directly slows down the generation of pigment substances that cause red and yellow changes [57]. The synergistic effect of these two mechanisms enables the GO + TG group to maintain the fresh color of the product to the greatest extent. The color difference analysis is consistent with the results of Section 3.1 and Section 3.2.

### 3.10. Molecular Dynamics Simulation Results

In simulation systems, overall structural stability is typically assessed using the root mean square deviation (RMSD). The radius of gyration (Rg) reflects the compactness of the three-dimensional conformation, while the root mean square fluctuation (RMSF) characterizes the local residue mobility. The number of hydrogen bonds (HB) indicates the strength of intermolecular interactions.

**Figure 10:**
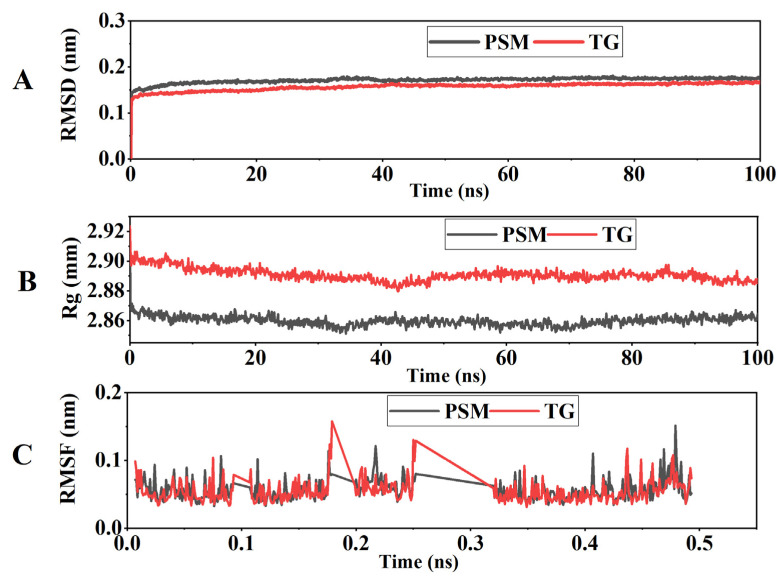
Molecular dynamics simulation results of pure soy protein meat (PSM) and tamarind polysaccharide gum (TG). (**A**) Root mean square deviation (RMSD); (**B**) Radius of gyration (Rg); (**C**) Root mean square fluctuation (RMSF). Note: PSM and TG, respectively, represent pure soybean meat and tamarind gum (*p* < 0.05).

The simulation results delineate the mechanism by which TG stabilizes the protein structure. The lower and more stable RMSD trajectory of the TG complex (0.148 ± 0.010 vs. 0.159 ± 0.010 for PSM) signifies suppressed global structural fluctuations, a effect attributable to TG’s steric hindrance (Figure 10A). This hindrance also resulted in a more consistent structural compactness, as reflected by the tighter Rg distribution of the complex (2.905 ± 0.013 nm) compared to the pure protein (2.89 ± 0.02 nm) (Figure 10B). At a local level, this stabilization manifested as a pronounced 20–30% decrease in residue fluctuations (RMSF) within flexible loop regions, directly demonstrating the restricted local mobility imposed by TG (Figure 10C). This indicates that the polysaccharide molecules effectively restricted local conformational changes by binding to specific sites on the protein surface.

**Figure 11:**
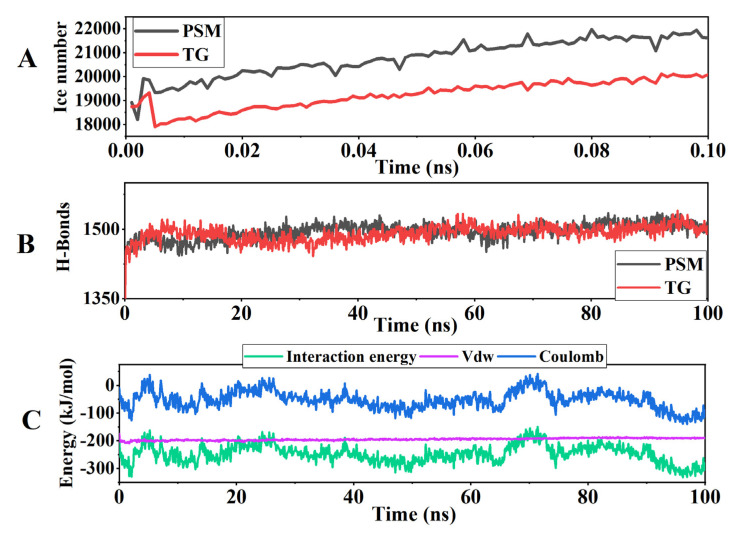
Molecular dynamics simulation results of pure soy protein meat (PSM) and tamarind polysaccharide gum (TG). (**A**) Ice number; (**B**) H-bones; (**C**) Energy changes. Note: PSM and TG, respectively, represent pure soybean meat and tamarind gum (*p* < 0.05). Vdw represents van der Waals forces, while Coulomb represents the electrostatic forces between molecules.

Notably, the number of hydrogen bonds (Figure 11B) in the complex system consistently remained within the range of 1400 to 1550, an increase of approximately 10% to 15% compared to the pure protein system (1043 to 1535). This directly proves the formation of a stable polar interaction network between tamarind gum and the protein. The ice number (Figure 11A) indicates that compared with PSM, the number of ice crystals in the TG system is lower and their growth is slower. This proves that TG can effectively inhibit the formation and growth of ice crystals, thereby reducing their mechanical damage to the protein network. The analysis of intermolecular forces shows that (Figure 11C) there are significant simultaneous van der Waals forces (Vdw) and Coulomb interactions between TG and soy protein. The Vdw provides stable close-range attraction, while the stronger electrostatic interaction reflects the complementarity of the TG with the surface charges of the protein. These two non-covalent interactions, together with hydrogen bonds, constitute a stable binding between TG and the protein. Through this molecular-level anchoring and spatial inhibition effect, the stability of the protein structure is enhanced, and the interface disturbance of ice crystals is indirectly inhibited. This explains the mechanism by which TG improves the freeze–thaw stability of plant meat at the atomic level.

As a neutral polysaccharide, TG’s abundant hydroxyl functional groups can form a dense hydrogen bond network with the polar residues on the protein surface [58]. This interaction not only enhances the rigidity of the protein structure but also maintains the moderately extended conformation of the protein through steric repulsion effects. Additionally, the hydration protective layer formed by the polysaccharide chains on the protein surface can reduce the disturbance of water molecules to the protein structure, further improving the stability of the system [59]. Research results confirm that the introduction of tamarind gum can effectively improve the structural stability of plant-based meat proteins through multiple molecular mechanisms, providing a theoretical basis and technical path for the development of high-quality plant-based foods.

## 4. Conclusions

This study successfully achieved its aim of developing a synergistic strategy to enhance the freeze–thaw stability of soy protein isolate (SPI)-based products by constructing a robust dual-network structure using glucose oxidase (GO) and tamarind gum (TG). The findings unequivocally demonstrate that the combination of GO and TG most effectively counteracted the detrimental effects of repeated freeze–thaw cycles, which include protein oxidation, aggregation, structural denaturation, and undesirable water migration. This synergistic action resulted in the optimal preservation of the product’s macroscopic quality, maintaining superior water-holding capacity, desirable texture, and minimal color changes compared to all other treatments. The underlying mechanism for this enhanced stability is attributed to the complementary roles of the two additives: GO strengthens the protein network internally through the enzymatic promotion of covalent disulfide bonds, while TG provides external physical stabilization via steric hindrance and enhanced water-binding capacity, collectively forming a more resilient and integrated matrix that can withstand freeze–thaw stresses.

To further strengthen these findings and advance their application, future research should validate this strategy in more complex and realistic food formulations containing lipids, flavors, and other ingredients and investigate the corresponding sensory properties to ensure consumer acceptance. From a commercial perspective, the use of GO and TG presents a highly promising natural and effective approach for improving the quality and shelf-life of plant-based meats. Given that both ingredients are commercially available and generally recognized as safe, their implementation is feasible. While a detailed cost–benefit analysis in an industrial context is warranted, the significant potential for reducing quality losses during frozen storage and distribution offers a compelling economic incentive, making this synergistic combination an attractive solution for manufacturers seeking to enhance the marketability and consumer satisfaction of their plant-based offerings.

## Figures and Tables

**Table 1:** The effect of 7 freeze–thaw cycles on the low-field nuclear magnetic resonance (LF-NMR) of plant-based meat.

Group	F-T Cycles	LF-NMR					
		Bound Water/T_21_	Non-Flowing Water/T_22_	Free Water/T_23_	Relative Area Response/P_21_ (%)	Relative Area Response/P_22_ (%)	Relative Area Response/P_23_ (%)
PSM	1	199.72 ± 1.99 ^a^	201.49 ± 1.67 ^a^	1.03 ± 0.15 ^b^	1035.32 ± 1.41 ^a^	2762.07 ± 47.02 ^c^	4.73 ± 0.73 ^c^
	3	181.51 ± 1.7 ^b^	191.52 ± 1.16 ^b^	1.51 ± 0.13 ^a^	874.82 ± 3.53 ^b^	3725.8 ± 0.83 ^a^	224.98 ± 0.47 ^b^
	5	166.01 ± 2.36 ^c^	173.29 ± 1.59 ^c^	0.87 ± 0.07 ^b^	799.38 ± 1.65 ^c^	2995.35 ± 1.57 ^b^	225.87 ± 0.74 ^ab^
	7	115.74 ± 0.75 ^d^	126.6 ± 1.82 ^d^	0.09 ± 0 ^c^	496.06 ± 1.48 ^d^	2636.72 ± 5.01 ^d^	227.02 ± 0.01 ^a^
PA	0	199.72 ± 1.99 ^a^	201.49 ± 1.67 ^a^	1.03 ± 0.15 ^b^	1035.32 ± 1.41 ^a^	2762.07 ± 47.02 ^c^	4.73 ± 0.73 ^c^
	1	130.26 ± 0.08 ^b^	140.76 ± 0.64 ^b^	3.47 ± 0.29 ^a^	881.96 ± 0.49 ^b^	3820.83 ± 14.87 ^a^	2.64 ± 0.04 ^d^
	3	113.12 ± 0.04 ^c^	120.27 ± 0.11 ^c^	1.3 ± 0.01 ^b^	881.05 ± 0.2 ^b^	3464.71 ± 2.27 ^b^	199.84 ± 0.74 ^b^
	5	110.25 ± 1.54 ^c^	118.99 ± 0.92 ^c^	1.29 ± 0.01 ^b^	880.31 ± 0.42 ^b^	3464.5 ± 4.49 ^b^	200.82 ± 0.71 ^ab^
	7	110.28 ± 0.05 ^c^	119.33 ± 0.02 ^c^	1.05 ± 0.06 ^b^	880.19 ± 0.04 ^b^	3416.89 ± 0.49 ^b^	201.91 ± 0.99 ^a^
TG	0	199.72 ± 1.99 ^a^	201.49 ± 1.67 ^a^	1.03 ± 0.15 ^cd^	1035.32 ± 1.41 ^a^	2762.07 ± 47.02 ^d^	4.73 ± 0.73 ^d^
	1	102.33 ± 1.43 ^b^	109.81 ± 0.71 ^b^	2.34 ± 0.01 ^a^	901.78 ± 0.8 ^c^	4031.98 ± 0.94 ^a^	1.14 ± 0.03 ^e^
	3	89.26 ± 0.07 ^c^	90.19 ± 0.04 ^c^	1.24 ± 0.04 ^b^	892.71 ± 0.71 ^d^	3620.29 ± 1.48 ^b^	123.6 ± 0.06 ^c^
	5	88.98 ± 0.05 ^c^	89.46 ± 0.21 ^c^	1.2 ± 0.01 ^bc^	901.71 ± 0.71 ^c^	3542.73 ± 28.94 ^c^	125.24 ± 0.04 ^b^
	7	87.66 ± 0.49 ^c^	88.22 ± 0.13 ^c^	1.02 ± 0.01 ^d^	905.67 ± 0.49 ^b^	3509.17 ± 3.19 ^c^	127.75 ± 0.79 ^a^
GO	0	199.72 ± 1.99 ^a^	201.49 ± 1.67 ^a^	1.03 ± 0.15 ^b^	1035.32 ± 1.41 ^a^	2762.07 ± 47.02 ^c^	4.73 ± 0.73 ^b^
	1	109.81 ± 0.71 ^b^	127.68 ± 3.76 ^b^	1.64 ± 0.4 ^a^	889.27 ± 1.49 ^b^	3860.18 ± 50.96 ^a^	2.24 ± 0.11 ^c^
	3	91.74 ± 0.81 ^c^	111.29 ± 1.49 ^c^	0.87 ± 0.04 ^b^	886.81 ± 0.71 ^b^	3380.59 ± 214.14 ^b^	180.83 ± 0.69 ^a^
	5	89.26 ± 1.34 ^c^	109.63 ± 0.54 ^c^	0.84 ± 0.04 ^b^	861.84 ± 3.58 ^c^	3461.11 ± 8.53 ^b^	181.83 ± 0.69 ^a^
	7	89.35 ± 0.96 ^c^	109.58 ± 0.52 ^c^	0.78 ± 0.01 ^b^	850.31 ± 1.41 ^d^	3458.24 ± 47.98 ^b^	182.14 ± 1.17 ^a^
GO + TG	0	199.72 ± 1.99 ^a^	201.49 ± 1.67 ^a^	1.03 ± 0.15 ^b^	1035.32 ± 1.41 ^a^	2762.07 ± 47.02 ^c^	4.73 ± 0.73 ^d^
	1	103.93 ± 0.45 ^b^	105.31 ± 1.41 ^b^	1.38 ± 0.04 ^a^	990.81 ± 0.71 ^b^	4226.28 ± 14.04 ^a^	1.28 ± 0.01 ^e^
	3	87.26 ± 0.07 ^c^	79.22 ± 0.13 ^c^	0.57 ± 0 ^c^	978.82 ± 0.7 ^c^	4030.76 ± 2.05 ^b^	109.86 ± 0.74 ^a^
	5	86.3 ± 0.02 ^c^	79.25 ± 0.06 ^c^	0.53 ± 0.01 ^c^	977.61 ± 0.57 ^c^	4012.91 ± 0.99 ^b^	102.91 ± 0.99 ^b^
	7	86.33 ± 0.19 ^c^	79.03 ± 0.03 ^c^	0.51 ± 0.03 ^c^	976.96 ± 0.07 ^c^	4006.07 ± 1.21 ^b^	101.23 ± 0.02 ^c^

Note: PSM, PA, TG, and GO, respectively, represent pure soybean meat, phytase, tamarind gum, and glucose oxidase. Values are mean ± SD; different superscript letters within a column indicate significant differences (*p* < 0.05).

## Data Availability

The original contributions presented in this study are included in the article. Further inquiries can be directed to the corresponding author.
